# A systematic review and meta-analysis of direct anterior approach versus posterior approach in total hip arthroplasty

**DOI:** 10.1186/s13018-018-0929-4

**Published:** 2018-09-06

**Authors:** Zhao Wang, Jing-zhao Hou, Can-hua Wu, Yue-jiang Zhou, Xiao-ming Gu, Hai-hong Wang, Wu Feng, Yan-xiao Cheng, Xia Sheng, Hong-wei Bao

**Affiliations:** From the department of orthopaedics, Jingjiang People’s Hospital, 28 No, Zhongzhou Road, Jingjiang, Taizhou City, 214500 Jiangsu Province China

**Keywords:** Direct anterior approach, Posterior approach, Total hip arthroplasty, Meta-analysis

## Abstract

**Background:**

This meta-analysis aimed to evaluate the postoperative clinical outcomes and safety of the direct anterior approach (DAA) versus posterior approach (PA) in total hip arthroplasty (THA).

**Methods:**

We searched PubMed, Embase, Web of Science, the Cochrane Library, and Google databases from inception to June 2018 to select studies that compared the DAA and PA for THA. Only randomized controlled trials (RCTs) were included. Outcomes included Harris hip score at 2 weeks, 6 weeks, 12 weeks, and 1 year; VAS at 24 h, 48 h, and 72 h; incision length, operation time, postoperative blood loss, length of hospital stay, and complications (intraoperative fracture, postoperative dislocation, heterotopic ossification (HO), and groin pain).

**Results:**

Nine RCTs totaling 754 THAs (DAA group = 377, PA group = 377) met the criteria to be included in this meta-analysis. The present meta-analysis indicated that, compared with PA group, DAA group was associated with an increase of the Harris hip score at the 2-week and 4-week time points. No significant difference was found between DAA and PA groups of the Harris hip scores at 12 weeks, 1 year length of hospital stay (*p* > 0.05). DAA group was associated with a reduction of the VAS at 24 h, 48 h, and 72 h with statistical significance (*p* < 0.05). What is more, DAA was associated with a reduction of the incision length and postoperative blood loss (*p* < 0.05). There was no significant difference between the operation time and complications (intraoperative fracture, postoperative dislocation, HO, and groin pain).

**Conclusion:**

In THA patients, compared with PA, DAA was associated with an early functional recovery and less pain scores. What is more, DAA was associated with shorter incision length and blood loss.

## Introduction

Total hip arthroplasty (THA) is an effective surgery alternative for patients with hip osteoarthritis (OA) or femoral head necrosis [1, 2]. Kurtz et al. [3] reported a 50% increase in the prevalence of THA from 1990 to 2002 and estimated nearly 572,000 THAs in 2030. Most THA patients experience pain relief, improved function, and restoration of quality of life [4]. However, nearly 7–15% patients were dissatisfied with THA due to the postoperative pain and functional recovery [5, 6]. The potential causes of postoperative pain include failure of fixation and damage of soft tissues [7]. Among the causes of damage of soft tissues, surgical approach was one of the influential factors [8, 9]. Choosing the optimal surgical approach could minimize pain severity, improve hip function, and thus increase patients’ satisfaction.

Currently, there are two common surgical approaches; direct anterior approach (DAA) and posterior approach (PA) are utilized in THA’s [10, 11]. Several reports have shown that the DAA was superior to the PA in terms of the postoperative blood loss and faster rehabilitation. The reason may be that DAA results in less soft tissue damage due to the fact that DAA relies on an intermuscular plane for insertion of the components [9]. For the above reasons, DAA has gained popularity in recent years [12]. However, some studies reported that DAA has more complications (femoral neck fracture, femoral perforation) than other approaches. Additionally, the learning curve of DAA has been reported to be relatively longer than other approaches [13, 14]. Two relevant meta-analyses [11, 15] that compare DAA with other approaches were published. However, shortcoming of these two meta-analyses should be noted. Higgins et al. [11] included retrospective studies and found that there was no significant difference between DAA and PA groups in the functional outcomes. Miller et al. [15] conducted a meta-analysis that compares DAA and PA for THA patients. However, they mixed the different follow-up outcomes for analysis and thus the heterogeneity was large. Another limitation was that they also included retrospective studies and thus selection bias could not be avoided.

Therefore, we conducted a meta-analysis based only on randomized controlled trials (RCTs) to compare the clinical outcomes of DAA versus PA in THA. We hypothesized that DAA is superior to PA in terms of the clinical outcomes in THA.

## Methods

This systematic review fully adhered to the preferred reporting items for systematic reviews and meta-analyses (PRISMA) guidelines [16].

### Search strategy

We manually searched PubMed, Embase, Web of Science, the Cochrane Library, and Google databases from inception to June 2018. There was no language restriction for all of the publications. The search terms included key words and Medical Subject Headings (MeSH) terms related to ““Arthroplasty, Replacement, Hip”[Mesh]”; total hip arthroplasty; total hip replacement; THA, THR, direct anterior approach, DAA, posterior approach, and PA. The reference lists of included studies or meta-analysis were also manually examined for potential missing records. This meta-analysis did not involve direct contact with individual patients; therefore, no ethics approval was needed.

### Inclusion criteria and exclusion criteria

1. Participants: patients prepared for THA.
2. Interventions: the intervention group received the DAA for THA.
3. Comparisons: the control group received PA for THA.
4. Outcomes: Harris hip score at 2 weeks, 6 weeks, 12 weeks, and 1 year; VAS at 24 h, 48 h, and 72 h; incision length, operation time, postoperative blood loss, length of hospital stay, and complications (intraoperative fracture, postoperative dislocation, heterotopic ossification (HO), and groin pain).
5. Study design: RCTs were regarded as eligible in the study.

Non-RCTs, letters, and editorial comments were excluded in this meta-analysis.

### Study selection

According to the formulated search strategy, all papers were guided into Endnote X7 (Thompson Reuters, CA, USA). Two reviewers (Zhao Wang and Hong-wei Bao) independently scanned the titles and abstracts of the potential studies. If there was a controversy between the reviewers, we asked a senior reviewer to make a decision.

### Date extraction

Two reviewers (Zhao Wang and Jing-zhao Hou) independently extract the following information: first author name and publication year, country, patients’ general characteristic (no. of patients, age, proportion of female patients, BMI), outcomes, study, and follow-up duration. The primary outcomes were Harris hip score at 2 weeks, 6 weeks, 12 weeks and 1 year, VAS at 24 h, 48 h, and 72 h; incision length, operation time, postoperative blood loss, length of hospital stay, and complications (intraoperative fracture, postoperative dislocation, heterotopic ossification (HO), and groin pain)**.**

### Outcome measures and statistical analysis

Continuous outcomes (Harris hip score at 2 weeks, 6 weeks, 12 weeks, and 1 year; VAS at 24 h, 48 h, and 72 h; incision length, operation time, postoperative blood loss, and length of hospital stay) were expressed as the weighted mean differences (WMD) with 95% confidence intervals (CIs). Complications (intraoperative fracture, postoperative dislocation, HO, and groin pain) were expressed as risk ratio (RR) with 95% CIs. *p* < 0.05 was considered statistically significant difference. Statistical analysis was performed using Stata software, version 12.0 (Stata Corp., College Station, TX, USA). To assess the heterogeneity, the *I*^2^ index and corresponding *p* value were calculated. When *I*^2^ was less than 50%, there was low heterogeneity; otherwise, there was a high heterogeneity. Publication bias was visually assessed using funnel plots (effect size was symmetry = no publication bias) and was quantitatively assessed using Begg’s test (*p* > 0.05 = no publication bias).

## Results

### Search results and general characteristic

**Fig. 1:**
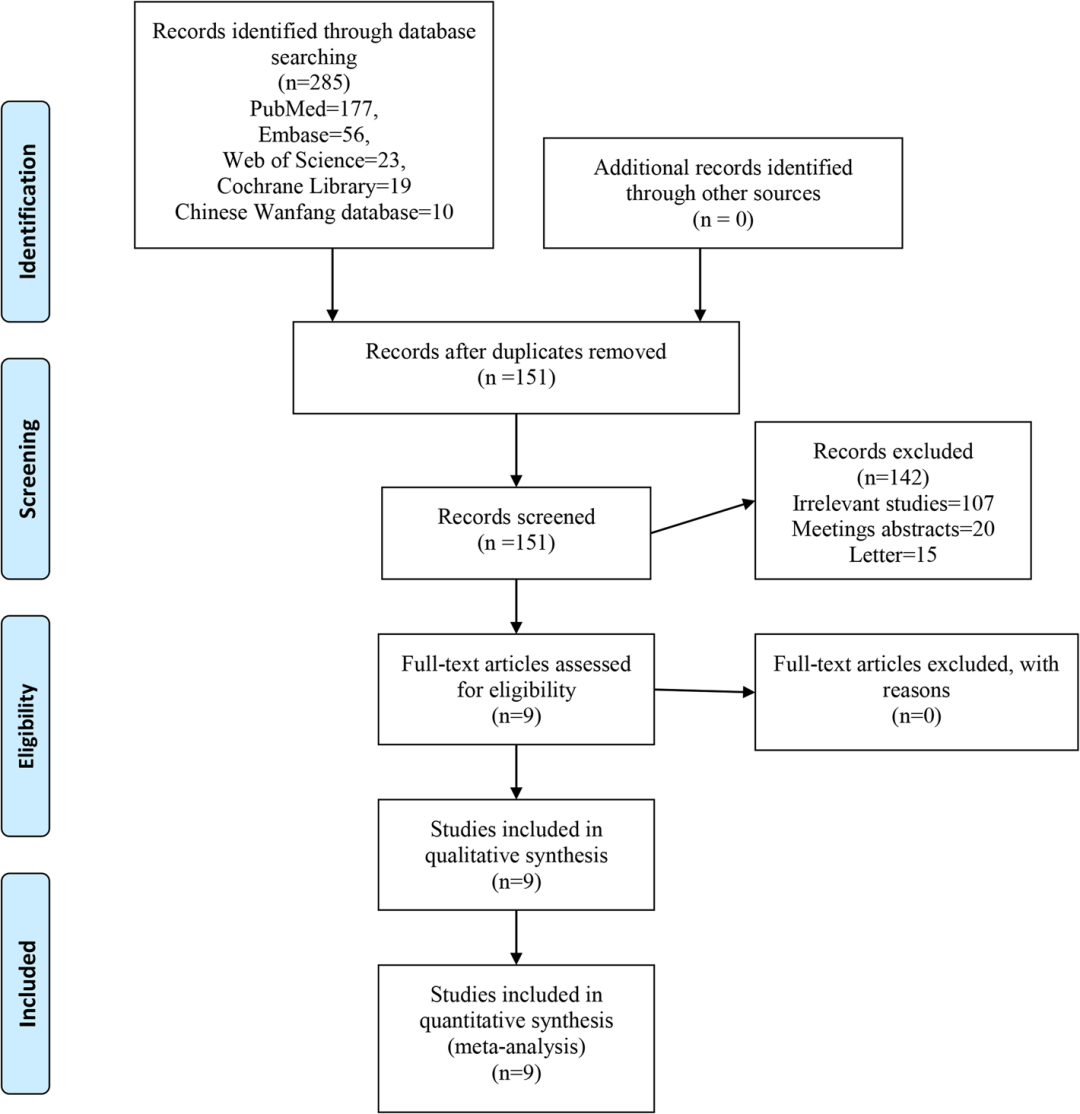
The flow diagram of study selection

Figure 1 shows the flowchart for selection of studies. First, a total of 285 studies were identified from the electronic databases (PubMed = 147, Embase = 56, Web of Science = 23, Cochrane Library = 19, Google database = 30). Then, all papers were input into Endnote X7 (Thomson Reuters Corp., USA) software for the removal of duplicate papers. A total of 151 papers were reviewed, and 142 papers were removed according to the inclusion criteria at abstract and title levels. Additionally, one study was a duplicate publication so we only included the most recently published paper. Ultimately, 9 clinical studies with 754 patients (DAA group = 377, PA group = 377) were involved in the meta-analysis [17–25]. The general characteristic of the included studies can be seen in Table 1. Publication years ranged from 2006 to 2018. Number of patients ranged from 27 to 60, and mean age ranged from 59 to 65. Follow-up duration ranged from 1 month to 1 year.

**Table 1:** General characteristic of the included studies

Author	No. of patients	Mean age (years)	Female (%)	BMI	Outcomes	Study	Follow-up
Barrett 2013^1,^^2,^^3,^^5,^^7^	43/44	61/63	33/57	31/29		RCT	3 months
Bergin 2011^2,^^4,^^8,^^9,^^10,^^11,^^12^	29/28	69/65	68/50	26/28		RCT	1 month
Christensen 2015^1,^^5,^^8,^^9,^	28/23	64/65	54/52	31/30		RCT	42 days
Rodriguez 2014^2,^^3,^^5,^^6^	60/60	59/60	34/32	28/24		RCT	1 year
Taunton 2014^1,^^3,^^4,^^8,^^10^	27/27	62/66	56/52	28/29		RCT	42 days
Cheng 2017^2,^^3,^^4,^^10,^^11,^^12^	35/27	59/63	57/53	28/28		RCT	84 days
Zhang 2006^1,^^2,^^5,^^8,^^10,^^12^	60/60	61/63	58/53	NS		RCT	3 months
Zhao 2017^2,^^3,^^5,^^6,^^8,^^9,^^11^	60/60	65/62	60/56	24/26		RCT	3 months
Zhang 2018^1,^^2,^^4,^^5,^^6,^^7,^	35/48	NS	NS	26/25		RCT	6 months

*NS*, not stated; *RCT*, randomized controlled trials; 1 Harris hip score at 2 weeks, 2, Harris hip score at 6 weeks, 3, Harris hip score at 12 weeks, 4 Harris hip score at 1 year, 5, VAS at 24 h, 6, VAS at 48 h, 7, VAS at 72 h, 8 incision length, 9, operation time, 10. postoperative blood loss, 11 length of hospital stay, 12 complications (intraoperative fracture, postoperative dislocation, heterotopic ossification (HO) and groin pain)

### Quality assessment

**Fig. 2:**
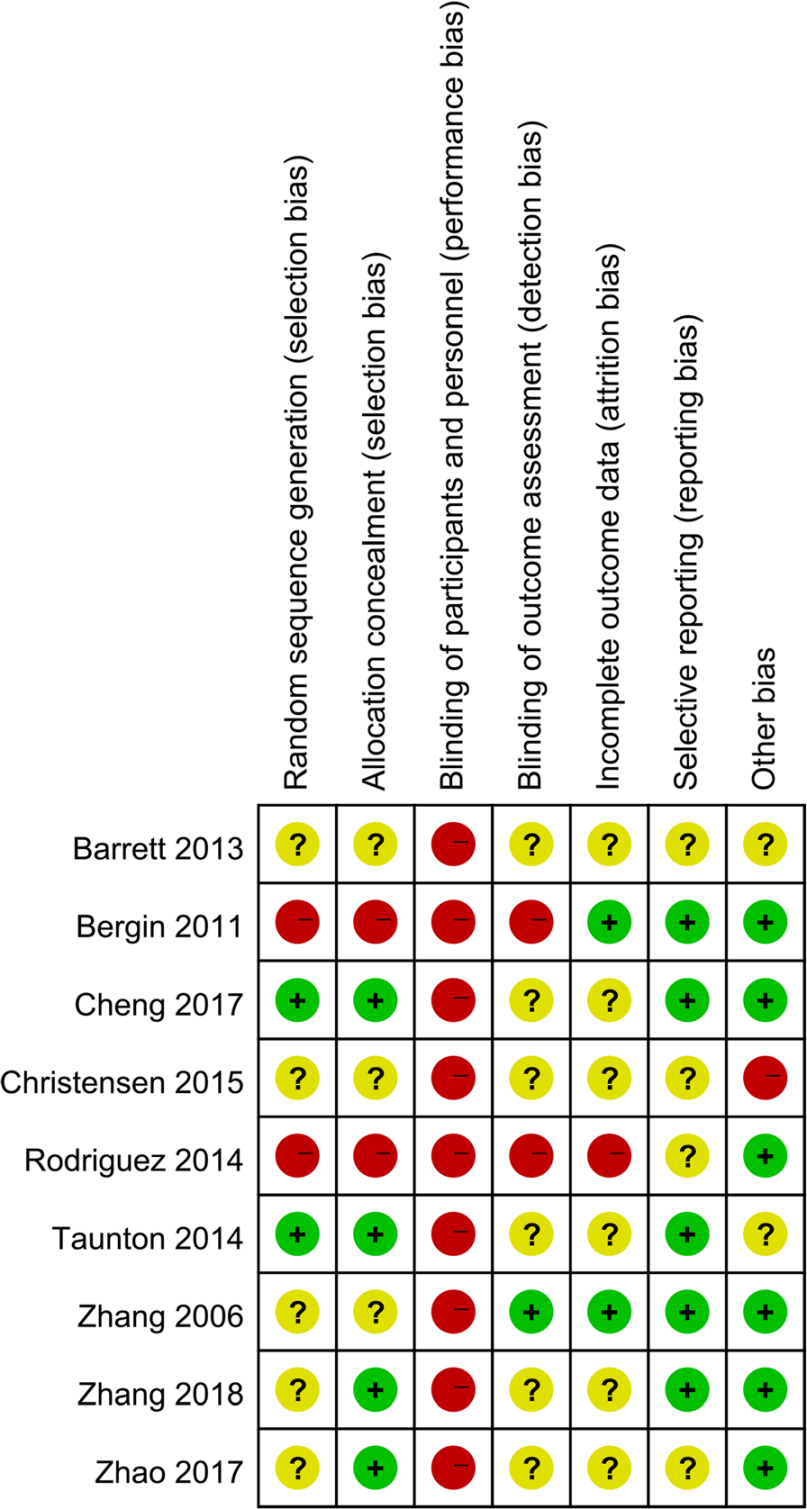
Risk of bias graph: review authors’ judgments about each risk of bias item presented as percentages across all included studies

**Fig. 3:**
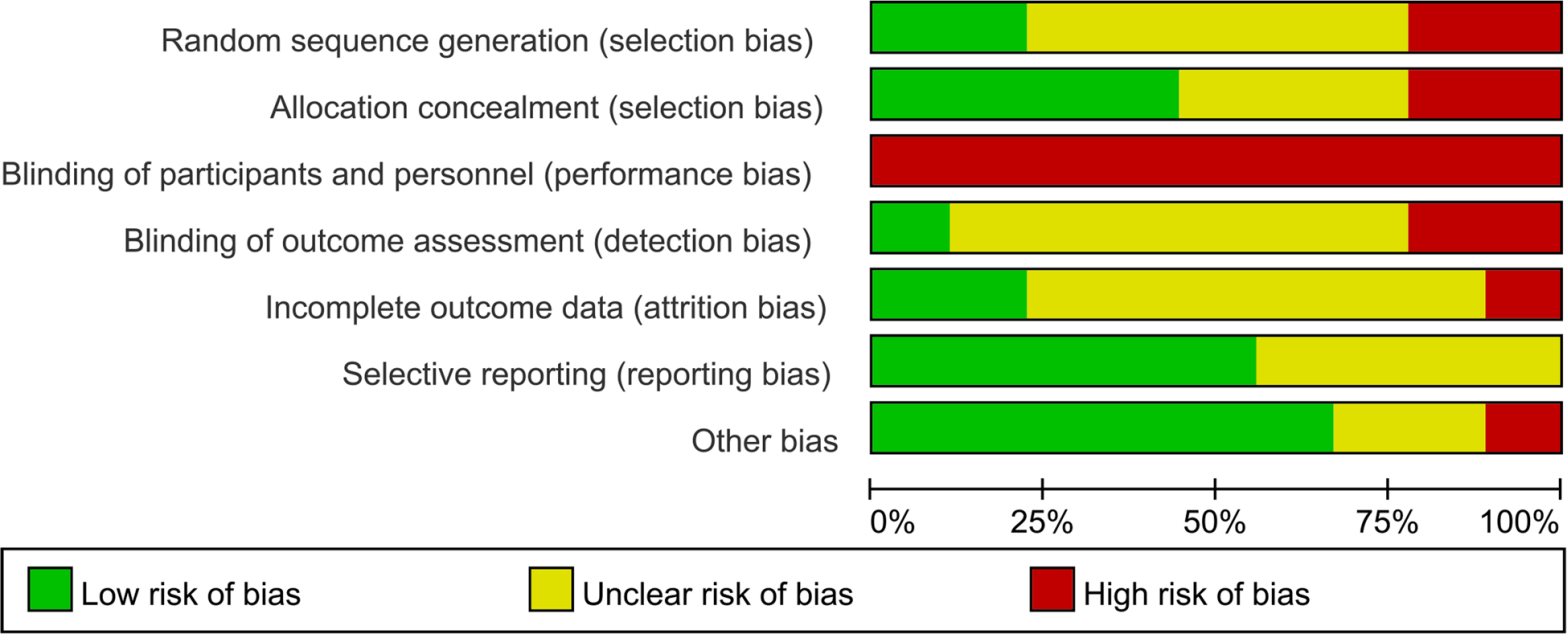
Risk of bias summary for included studies. +, no bias; −, bias;?, bias unknown

The risk of bias graph and risk of bias summary is summarized in Figs. 2 and 3 respectively. Random sequence generation procedure (selection bias) was low and unclear in two and five of the included studies respectively. Allocation concealment was low in four studies and high in two studies. Blinding of participant was with high risk of bias in all of the included studies. Attrition bias was unclear in six studies. Other bias was high in one study and two were with unclear risk of bias, the rest were all with low risk of bias.

### Results of meta-analysis

#### Harris hip score at 2 weeks, 6 weeks, 12 weeks, and 1 year

**Fig. 4:**
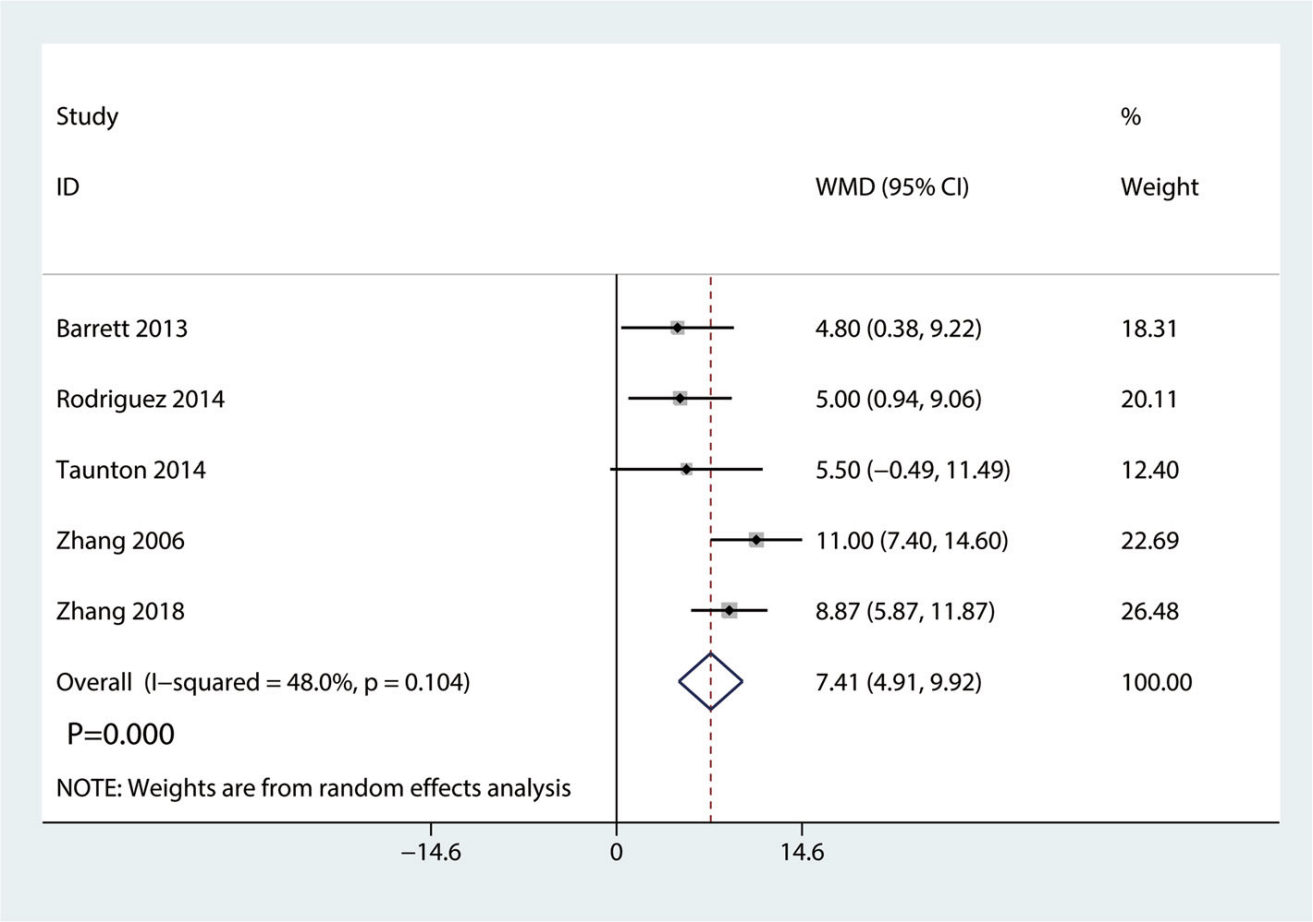
Forest plot for comparing DAA versus PA in terms of Harris hip score at 2 weeks

**Fig. 5:**
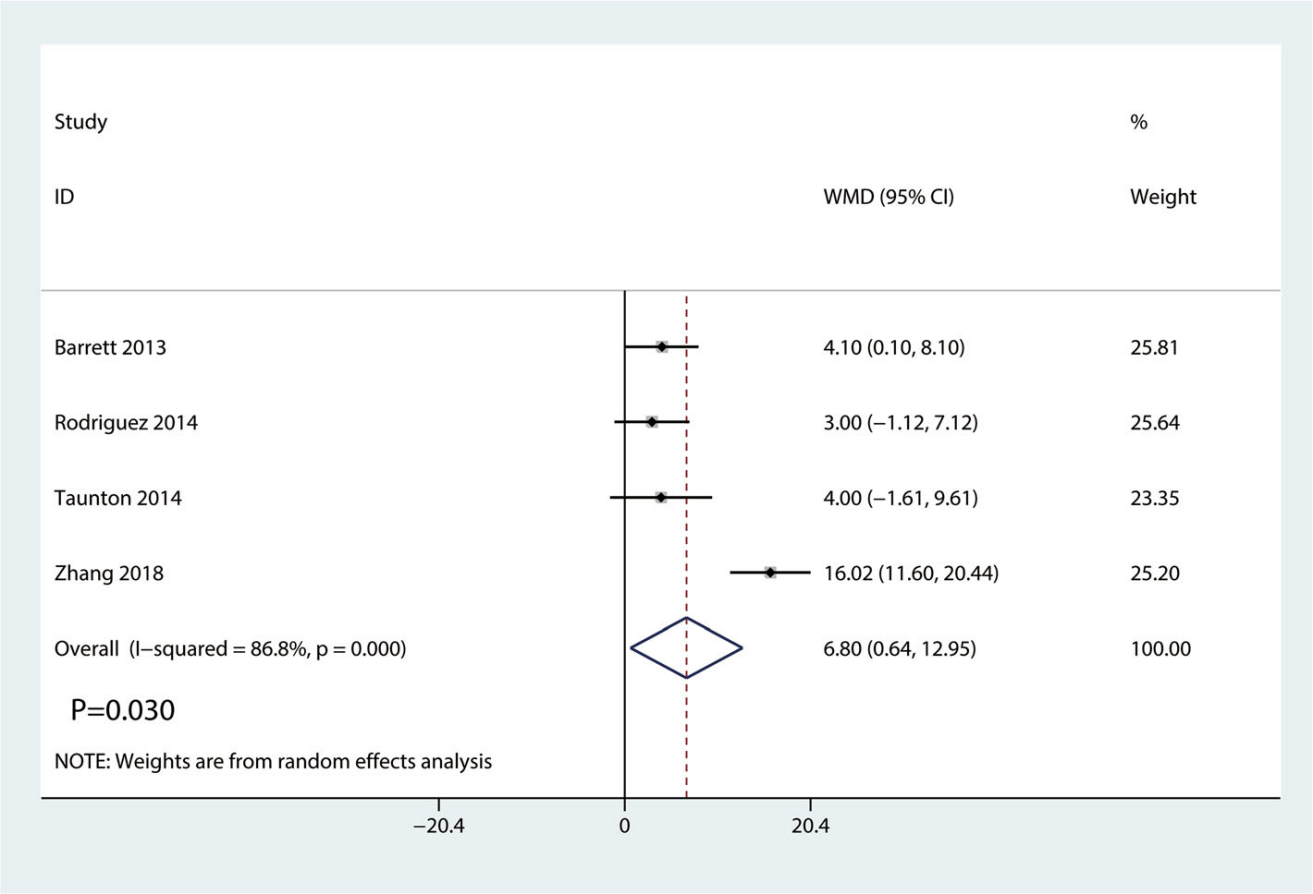
Forest plot for comparing DAA versus PA in terms of Harris hip score at 6 weeks

**Fig. 6:**
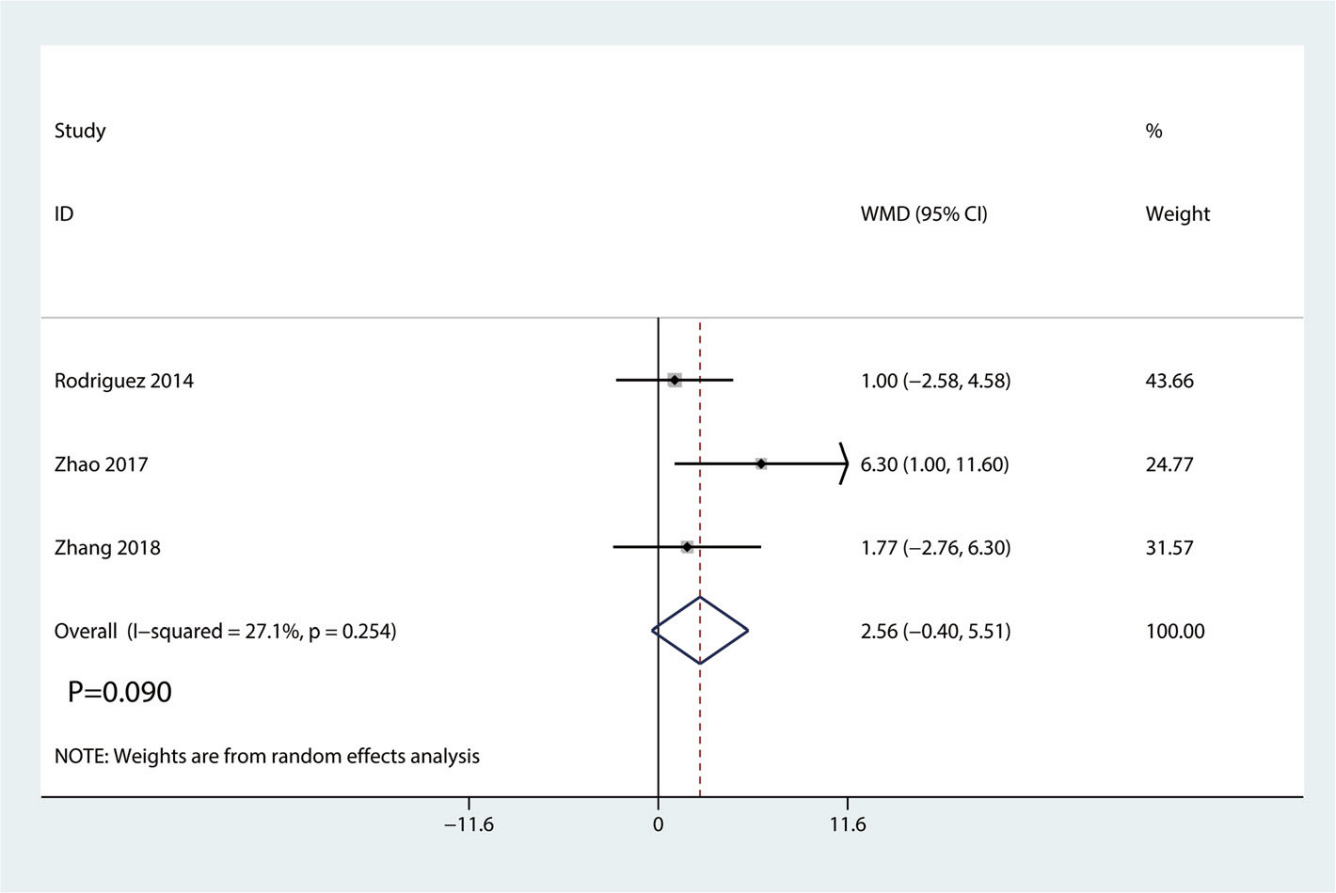
Forest plot for comparing DAA versus PA in terms of Harris hip score at 12 weeks

**Fig. 7:**
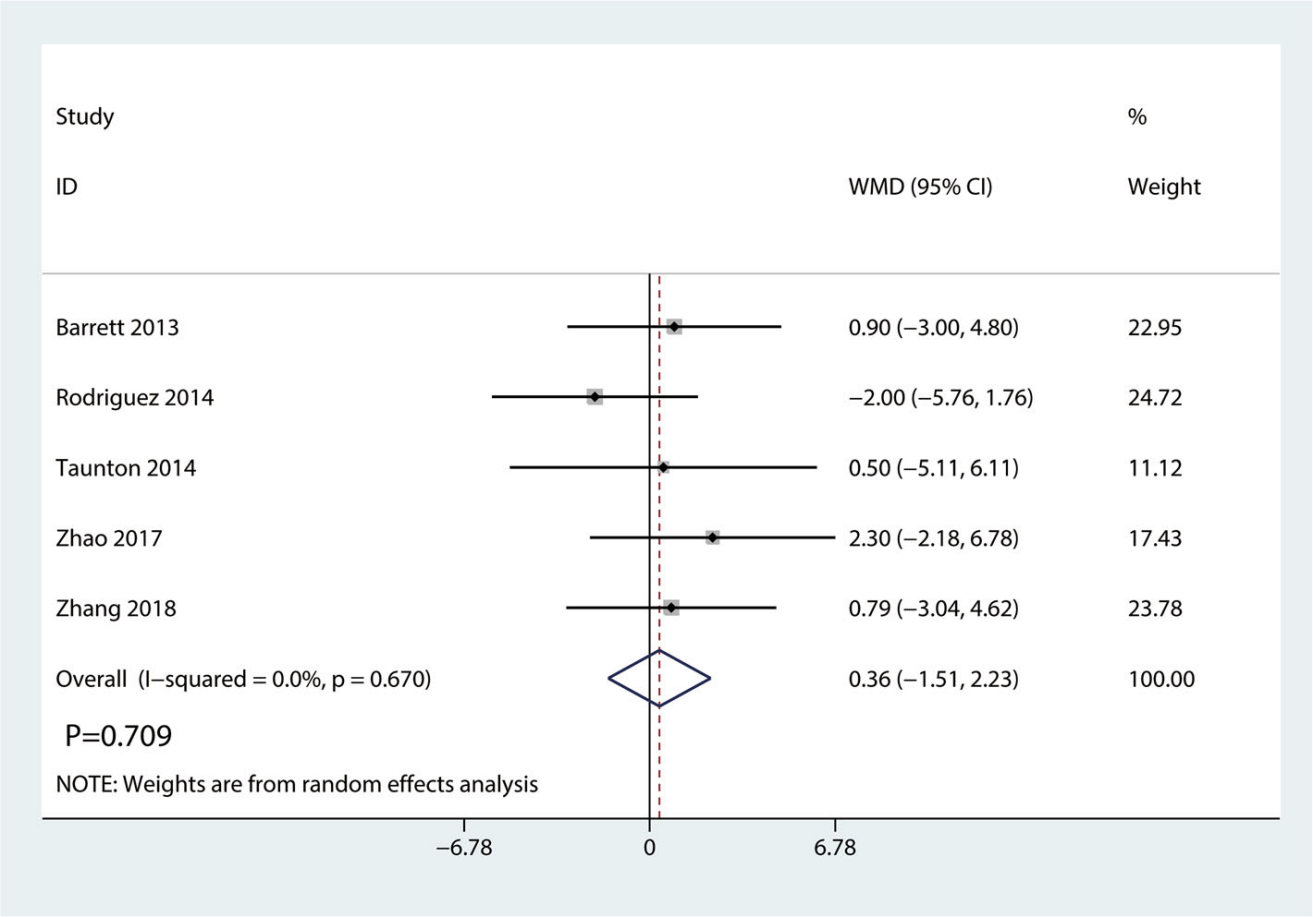
Forest plot for comparing DAA versus PA in terms of Harris hip score at 1 year

Data on 661 primary THAs (including 329 with DAA and 322 with PA) were pooled from 5 trials analyzing the Harris hip score at 2 weeks. The DAA group was associated with an increase of the Harris hip at 2 weeks and 6 weeks (2 weeks, WMD = 7.41, 95%CI 4.91 to 9.92, *p* = 0.000; Fig. 4; 6 weeks, WMD = 6.80, 95%CI 0.64 to 12.95, *p* = 0.030, Fig. 5). The DAA and PA groups were not statistically significantly different with regard to pain at 12 weeks and 1 year (12 weeks, WMD = 2.56, 95%CI − 0.40 to 5.51, *p* = 0.090, Fig. 6; 1 year, WMD = 0.36, 95%CI − 1.51 to 2.23, *p* = 0.709, Fig. 7).

#### VAS at 24 h, 48 h, and 72 h

**Fig. 8:**
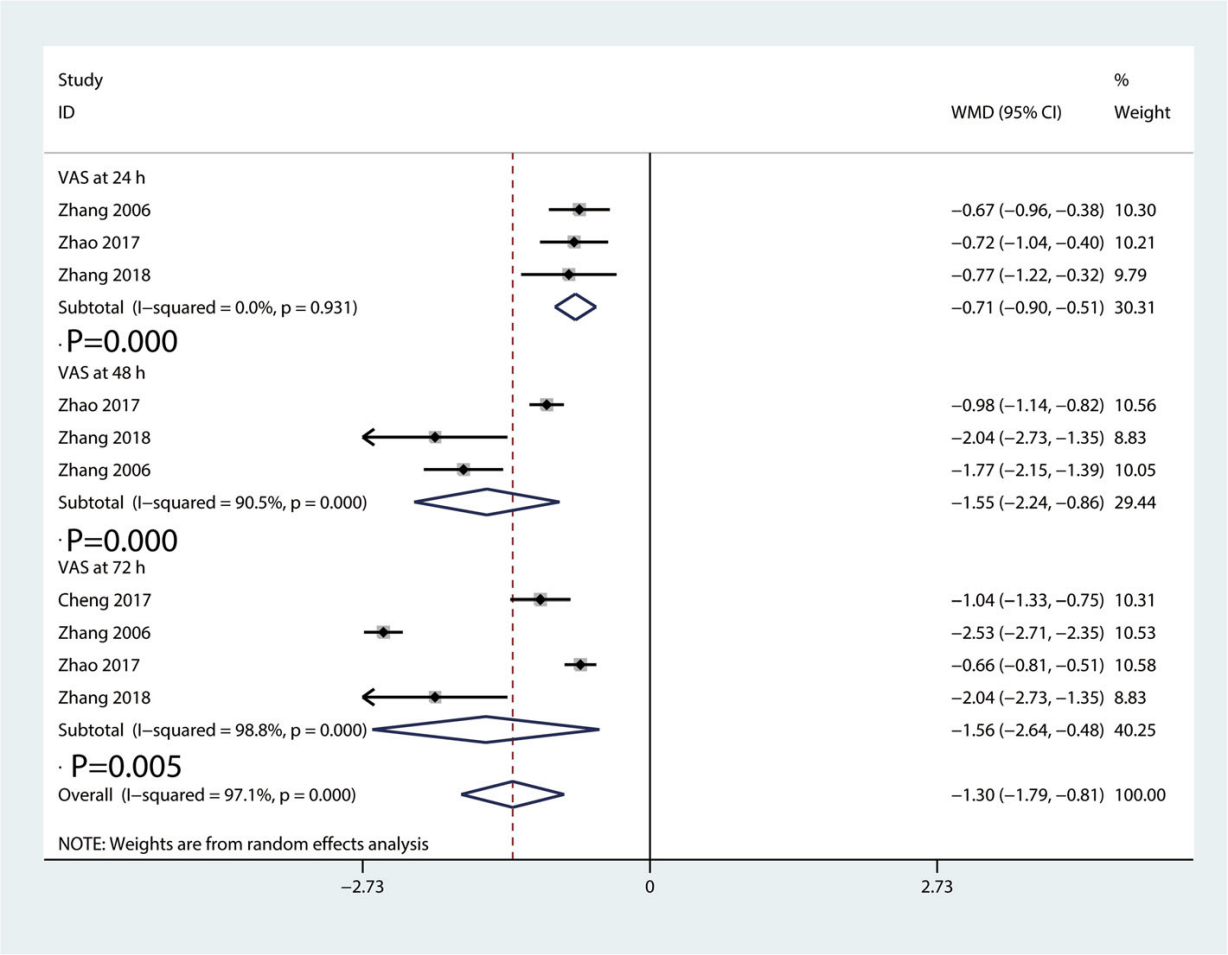
Forest plot for comparing DAA versus PA in terms of VAS at 24 h, 48 h, and 72 h

Compared with PA group, the DAA group was associated with a decrease of the VAS at each time point (24 h, WMD = − 0.71, 95%CI − 0.90 to − 0.51, *p* = 0.000; 48 h, WMD = − 1.55, 95%CI − 2.24 to − 0.86, *p* = 0.000; 72 h, WMD = − 1.56, 95%CI − 2.64 to − 0.48, *p* = 0.005, Fig. 8).

#### Incision length

**Fig. 9:**
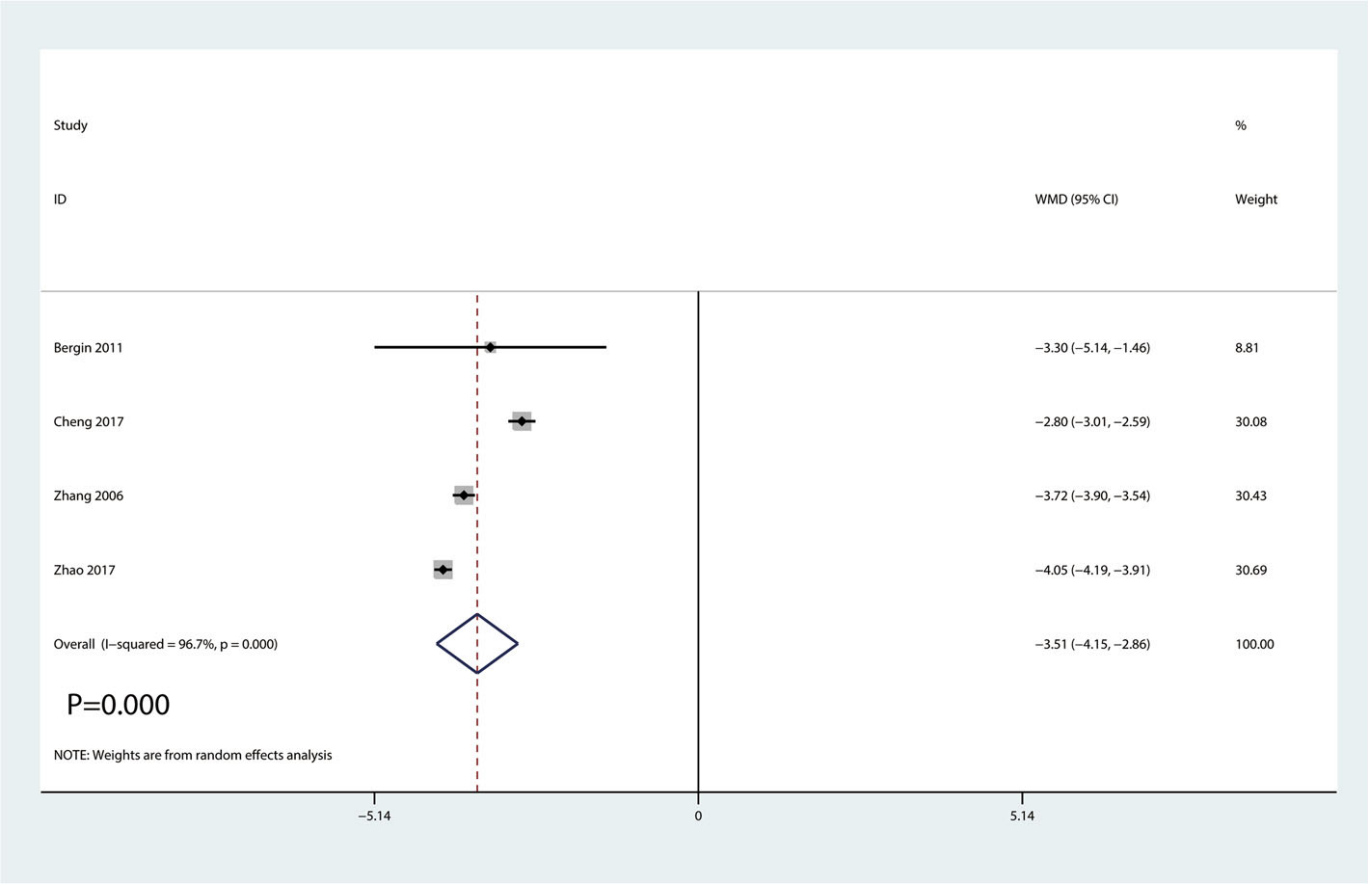
Forest plot for comparing DAA versus PA in terms of incision length

Data on 359 primary THAs (including 184 with DAA and 175 with PA) were pooled from 4 trials analyzing the incision length. Compared with PA, DAA group was associated with a reduction of the incision length by 3.51 cm (WMD = − 3.51, 95%CI − 4.15 to − 2.86, *p* = 0.000, Fig. 9).

#### Operation time

**Fig. 10:**
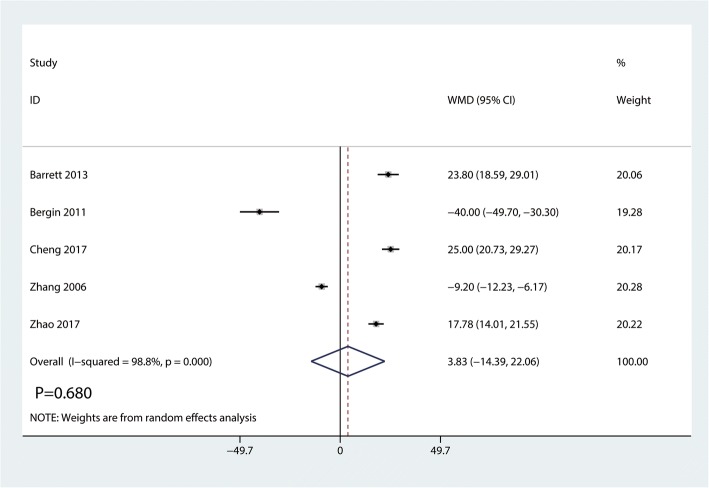
Forest plot for comparing DAA versus PA in terms of operation time

Data on 446 primary THAs (including 227 with DAA and 219 with PA) were pooled from 5 trials analyzing the operation time. Compared with PA, DAA group was not associated with an increase of the operation time (WMD = 3.83, 95%CI − 14.39 to 22.06, *p* = 0.680, Fig. 10).

#### Postoperative blood loss

**Fig. 11:**
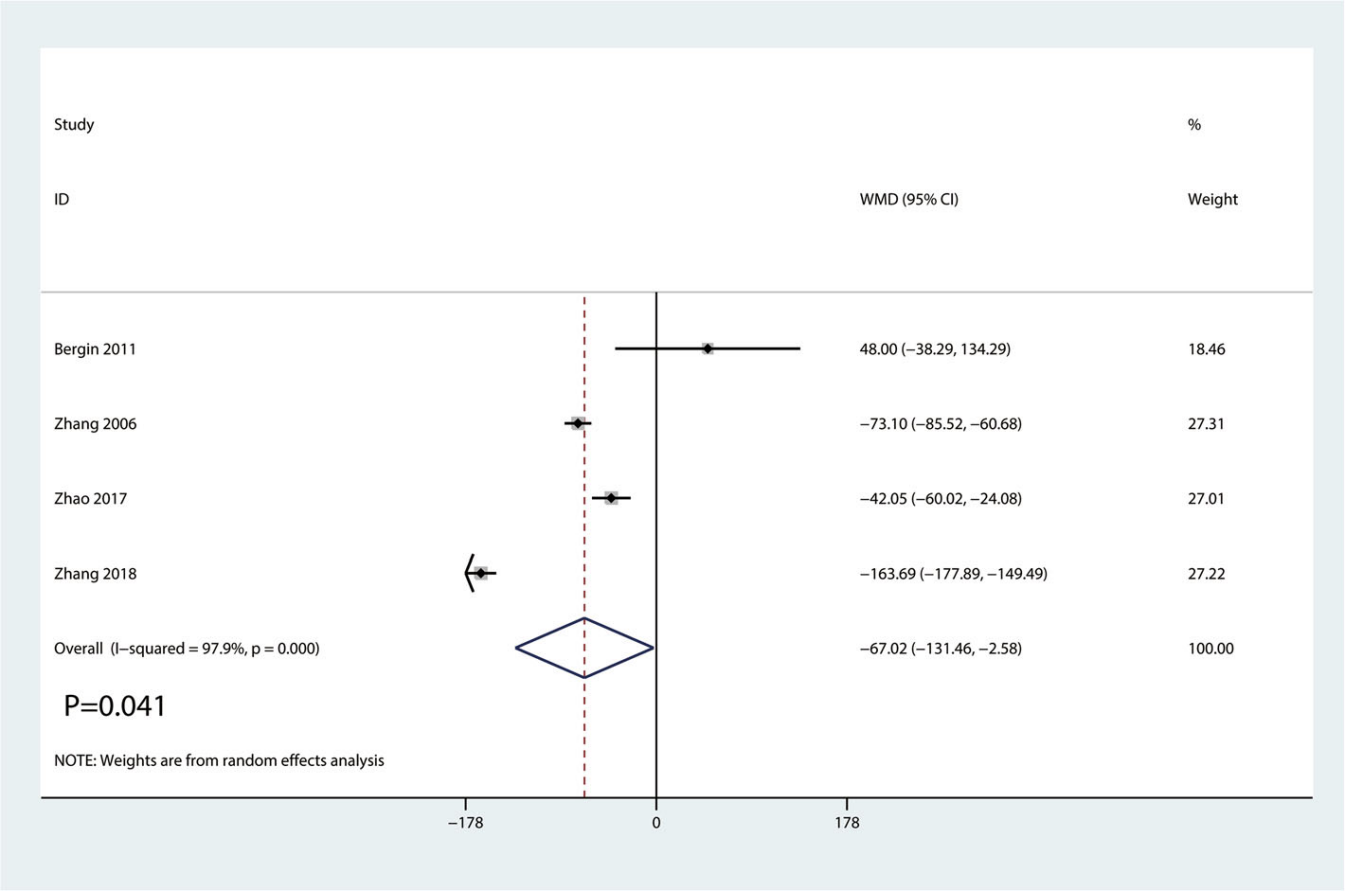
Forest plot for comparing DAA versus PA in terms of postoperative blood loss

Data on 380 primary THAs (including 184 with DAA and 196 with PA) were pooled from 4 trials analyzing the postoperative blood loss. Compared with PA, DAA group was associated with a reduction of the postoperative blood loss (WMD = − 67.02, 95%CI − 131.46 to − 2.58, *p* = 0.041, Fig. 11).

#### Length of hospital stay

**Fig. 12:**
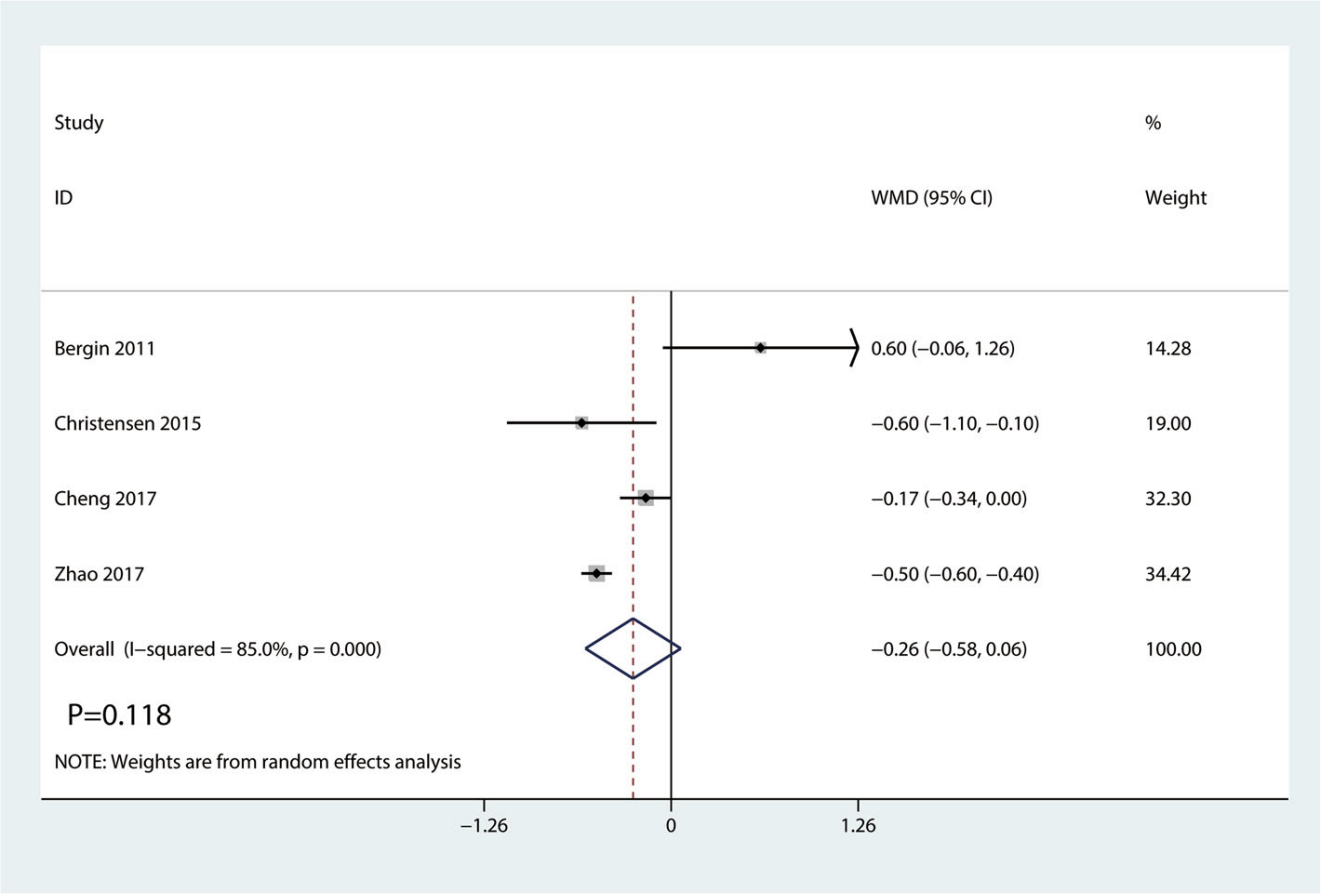
Forest plot for comparing DAA versus PA in terms of the length of hospital stay

Four studies totaling 290 THAs (DAA = 152, PA = 138) analyzing the length of hospital stay. There was no significant difference between the DAA group and PA group in terms of the length of hospital stay (WMD = − 0.26, 95%CI − 0.58 to 0.06, *p* = 0.118, Fig. 12).

#### Complications

**Fig. 13:**
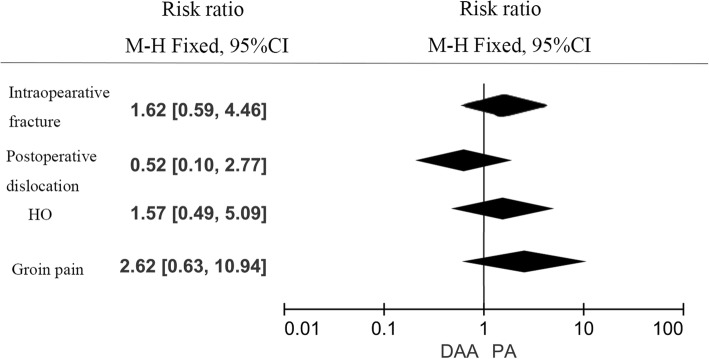
Forest plot for comparing DAA versus PA in terms of complications

There was no significant difference between DAA group and PA group in terms of the intraoperative fracture (RR = 1.62, 95%CI 1.62 to 4.46, *p* = 0.350, Fig. 13); postoperative dislocation (RR = 0.52, 95%CI 0.10 to 2.27, *p* = 0.441, Fig. 13), HO (RR = 1.57, 95%CI 0.49 to 5.09, *p* = 0.450, Fig. 13), and groin pain (RR = 2.62, 95%CI 0.63 to 10.94, *p* = 0.191, Fig. 13).

## Discussion

### Main findings

Our meta-analysis indicated that DAA has a positive role in reducing acute pain intensity, improving postoperative rehabilitation, and decreasing the length of incision and blood loss. We used sensitivity analysis to further confirm the reliability of our conclusion. Most of our analyses were low- to middle-quality evidence.

### New knowledge of this meta-analysis

A major strength of our current meta-analysis is that we limited the inclusion criteria to RCTs. Another new knowledge of this meta-analysis is that we performed a comprehensive analysis (postoperative hip function at same duration follow-up, postoperative pain intensity, blood loss, length of incision, and the length of hospital stay). As far as we know, this meta-analysis is the most comprehensive one to date to compare DAA versus PA for THA.

### Implications for clinical practice

We found statistically significant differences between DAA and PA with regards to Harris hip score at 2 weeks and 4 weeks post op. Putananon et al. [26] performed a network meta-analysis that compares DAA, lateral, PA, and posterior approaches in THA. Those results showed that DAA for THA gave the best postoperative VAS and Harris hip score. However, they only compared the VAS and Harris hip scores at final follow-up. In our current meta-analysis, we categorized the VAS and Harris hip score at multiple time intervals post-operatively. Our results showed that DAA was superior to PA in terms of the Harris hip score at 2 weeks and 6 weeks. There was no significant difference between the DAA and PA groups in terms of the Harris hip score at 12 weeks and 1 year follow-up. Zhao et al. [24] found that DAA group was associated with a better functional recovery than PA group at 3 months. However, there was no significant difference between DAA and PA groups at 6 month follow-up.

We also found that the DAA group was associated with a reduction of pain intensity at 24 h, 48 h, and 72 h compared to the PA group. One possible rationale for improving Harris hip score and decreasing pain intensity was the avoidance of muscle splitting and reduced soft tissue damage in DAA group than that of PA group. We found two RCTs that use C-reactive protein (CRP) level to support our hypothesis [24]. Zhao et al. [24] found that, for postoperative day 1 to 4, the level of CRP, IL-6, and ESR was significantly lower in DAA group than that in PA group.

We compared four complications (intraoperative fracture, postoperative dislocation, HO, and groin pain) between DAA group and PA group. Results showed that there was no significant difference between these complications (*p* > 0.05). Theoretically speaking, DAA has also been suggested to have an advantage in terms of dislocation risk over PA THA. Current meta-analysis found no significant difference between DAA and PA groups in terms of the postoperative dislocation. Two studies [20, 24] initiated after performance of 150 or 100 THAs via the direct anterior approach and thus could minimize the influence of a learning curve. The revision rate and risk of revision was comparable in DAA group and PA group in THA [27].

Several limitations in this meta-analysis should be noted. First, the follow-up duration of VAS was relatively short, and long-term follow-up is necessary to identify the long-term effects of DAA. Second, learning curve of the DAA and PA were not reported in the included studies and thus we cannot comment on the learning curve regarding either approach. Third, postoperative rehabilitation program was different and thus may cause heterogeneity for the final outcome. Lastly, the blinding of the participant was high in all of the studies, and this high bias affects the final outcomes.

## Conclusion

In THA patients, compared with PA, DAA was associated with an early functional recovery and lower pain scores. What’s more, DAA was associated with shorter incision length and blood loss. There was no significant difference in complication rated between the DAA and PA groups. Considering the limitation of this meta-analysis, more high quality RCTs are needed to further identify the effects of DAA in THA patients.

## Abbreviations

CIs: Confidence intervals
CRP: C-reactive protein
DAA: Direct anterior approach
HO: Heterotopic ossification
MeSH: Medical Subject Headings
OA: Osteoarthritis
PA: Posterior approach
PRISMA: preferred reporting items for systematic reviews and meta-analyses
RCT: Randomized controlled trials
RR: Risk ratio
THA: Total hip arthroplasty
WMD: Weighted mean difference
